# Key Physiological Parameters Dictate Triggering of Activity-Dependent Bulk Endocytosis in Hippocampal Synapses

**DOI:** 10.1371/journal.pone.0038188

**Published:** 2012-06-04

**Authors:** Eva M. Wenzel, Andrew Morton, Katrin Ebert, Oliver Welzel, Johannes Kornhuber, Michael A. Cousin, Teja W. Groemer

**Affiliations:** 1 Department of Psychiatry and Psychotherapy, University of Erlangen-Nürnberg, Erlangen, Germany; 2 Centre for Integrative Physiology, University of Edinburgh, Edinburgh, Scotland, United Kingdom; 3 Centre for Cancer Biomedicine, Faculty of Medicine, University of Oslo, Montebello, Oslo, Norway; The University of Queensland, Australia

## Abstract

To maintain neurotransmission in central neurons, several mechanisms are employed to retrieve synaptically exocytosed membrane. The two major modes of synaptic vesicle (SV) retrieval are clathrin-mediated endocytosis and activity-dependent bulk endocytosis (ADBE). ADBE is the dominant SV retrieval mode during intense stimulation, however the precise physiological conditions that trigger this mode are not resolved. To determine these parameters we manipulated rat hippocampal neurons using a wide spectrum of stimuli by varying both the pattern and duration of stimulation. Using live-cell fluorescence imaging and electron microscopy approaches, we established that stimulation frequency, rather than the stimulation load, was critical in the triggering of ADBE. Thus two hundred action potentials, when delivered at high frequency, were sufficient to induce near maximal bulk formation. Furthermore we observed a strong correlation between SV pool size and ability to perform ADBE. We also identified that inhibitory nerve terminals were more likely to utilize ADBE and had a larger SV recycling pool. Thus ADBE in hippocampal synaptic terminals is tightly coupled to stimulation frequency and is more likely to occur in terminals with large SV pools. These results implicate ADBE as a key modulator of both hippocampal neurotransmission and plasticity.

## Introduction

In synapses of the central nervous system as well as at the neuromuscular junction (NMJ) a fast mechanism of membrane retrieval following exocytosis of synaptic vesicles (SVs) is necessary for maintenance of neurotransmission. This is particularly important during intense stimulation, where fused SV membrane must be quickly retrieved to avoid excess plasma membrane growth and to regenerate vesicles for subsequent transmitter release.

At central synapses, different modes of compensatory endocytosis have been described. Clathrin-mediated-endocytosis (CME) is the dominant SV retrieval mode in central synapses during mild stimulation [1]. CME retrieves membrane by regenerating SVs directly from the plasma membrane by forming clathrin-coated pits that subsequently generate SVs by a dynamin-dependent process [2]. Kiss-and-run is a mechanism whereby SVs secrete their content via a small pore formed between the SV and the plasma membrane, after which the SV is retrieved intact. Kiss-and-run is an established mechanism of membrane retrieval in neurosecretory and mast cells [3], [4], [5], [6], [7], however its contribution at central synapses and neuromuscular junctions remains unclear (discussed in [8]). During high intensity stimulation a third mode of compensatory membrane retrieval is triggered, called activity-dependent bulk endocytosis (ADBE). ADBE is the dominant SV retrieval mode during intense stimulation [9] and compensates for plasma membrane increases that cannot be reversed in the short term by single vesicle endocytosis, due to capacity limitations of the CME machinery [10], [11], [12], [13]. SVs are generated from the resulting endosomal compartments and slowly refill the reserve pool [14], [15], [16]. Thus central nerve terminals possess a variety of SV retrieval strategies that allow them to adapt to a wide spectrum of neuronal activity.

ADBE has been reported in both peripheral and central synapses from various species [9], [13], [16], [17], [18], [19], [20], [21], [22]. However, its molecular mechanism is only starting to be determined [23]. Similarly, the physiology of ADBE is still poorly understood, with only a few studies investigating the relationship between ADBE and stimulation paradigms relevant to neuronal activity [9], [15], [21]. To directly address the relationship between ADBE and physiological stimulation, we performed a systematic investigation in primary cultures of rat hippocampal neurons. This experimental system was chosen since it is the most extensively employed for studies of basic SV recycling.

In these studies we manipulated neurons using a wide spectrum of stimuli by varying both the pattern and duration of stimulation. ADBE and SV recycling were monitored using a range of fluorescent fluid phase, lipophilic and antibody-based reporters. We established that the pattern of stimulation, rather than the stimulation load, was critical in the triggering of ADBE. Furthermore we determined that nerve terminals with larger SV recycling pools have a greater potential to undergo ADBE. We also discovered that inhibitory vGAT-positive nerve terminals had on average larger SV recycling pools and a higher likelihood to perform ADBE.

## Materials and Methods

### Ethics Statement

All animals were handled in strict accordance with good animal practice as defined by the guidelines of the State of Bavaria, and all animal work was approved by the Kollegiales Leitungsgremium of the Franz-Penzoldt Zentrum, Erlangen (reference number TS-1/10).

### Cell Culture

Hippocampal neuronal cultures were prepared from one to four days old Wistar rats (Charles River, USA) as described [24]. Newborn rats were sacrificed by decapitation. Hippocampi were removed from the brain in ice cold Hank’s salt solution, and the dentate gyrus was cut away. After digestion with trypsin (5 mg ml^−1^) cells were triturated mechanically and plated in MEM medium, supplemented with 10% fetal calf serum and 2% B27 Supplement (all from Invitrogen, Taufkirchen). If required, neurons were transfected after 3 days in vitro with synaptopHluorin [25] under control of a synapsin promoter with a modified calcium phosphate method as described [24]. Dextran/synaptotagmin experiments were performed between 22 and 25 days *in vitro* (DIV), vGAT-Oyster®488 experiments on DIV 32 and FM/synaptotagmin experiments on DIV 31.

### Imaging

Experiments were conducted at room temperature on a Nikon TI-Eclipse inverted microscope equipped with a 60×, 1.2 NA water immersion objective and Perfect Focus System™. Fluorescence was excited by a Nikon Intensilight C-HGFI through excitation filters centred at 482 nm, 561 nm and 628 nm. Emitted light passed dichroic longpass mirrors (cut-off wavelength 500 nm, 570 nm and 660 nm) prior to emission band-pass filters ranging from 500 nm –550 nm, 570 nm –640 nm and 660 nm –730 nm, respectively (Semrock, Rochester) and was projected onto a cooled EM-CCD camera (iXonEM DU-885 and iXonEM DU-897, Andor). Cover slips were placed into a perfusion chamber (vol  = 500 µl) containing saline (144 mM NaCl, 2.5 mM KCl, 2.5 mM CaCl_2_, 2.5 mM MgCl_2_, 10 mM Glucose, 10 mM Hepes, pH 7.5). Synaptic boutons were stimulated by electric field stimulation (platinum electrodes, 10 mm spacing, 1 ms pulses of 50 mA and alternating polarity); 10 µM 6-cyano-7-nitroquinoxaline-2,3-dione (CNQX, Tocris Bioscience) and 50 µM DL-2-Amino-5-phosphonopentanoic acid (DL-AP5, Tocris Bioscience) were added to prevent recurrent activity.

Recorded image stacks were used to automatically detect spots of synaptic bouton size by a Laplace-operator-based peak detection method [26]. ROI smaller than 5 or larger than 50 pixels were excluded, the threshold was defined as described [27]. All image and data analysis was performed using custom-written routines in MATLAB (The MathWorks Inc., Natick).

### Experimental Procedure

Cultured dispersed hippocampal neurons were incubated for 0.5 to 1 h with 0.6 µg of an CypHer^TM^5E labelled anti-synaptotagmin1 antibody that recognizes the luminal domain of synaptotagmin1 (αSyt1-cypHer5, Synaptic Systems, Goettingen) in saline (formulation as described above) to later identify synaptic boutons. Cover slips were then placed into a custom made perfusion chamber (vol  = 200 µl) containing saline. 40 kDa dextran tetramethylrhodamine (Invitrogen, Karlsruhe) was applied to achieve a final concentration of 50 µM and cells were subsequently stimulated by electric field stimulation of various intensities (platinum electrodes, 10 mm spacing, 1 ms pulses of 50 mA and alternating polarity). Non-internalized dextran tetramethylrhodamine was thoroughly washed away, and then images were acquired in the TRITC and the cy5 channel. Several fields of view (133 µm ×134 µm) were recorded from every cover slip.

FM1-43 staining was done By loading cells with 1 µM or 2.5 µM FM1-43 by electrical field stimulation with 1200 AP, 30 Hz. After test stimuli, complete unloading was performed by two subsequent electrical stimulations (900 AP/30 Hz) as described [28], [29]. dFM for every synaptic bouton was calculated as the difference between the mean initial fluorescence and the fluorescence intensity following the complete unloading. CypHer-labelled SytI-antibody fluorescence was quantified as the mean fluorescence of every synaptic terminal.

Inhibitory synapses were identified with αvGAT-Oyster®488, an antibody directed against the lumenal domain of the vesicular GABA transporter (Synaptic Systems, Goettingen) by incubating the neurons in saline for at least 3.5 h together with the αSyt1-cypHer5 (0.6 µg each).

In the synaptopHluorin experiments boutons were stimulated with 200 AP, 80 Hz or 1200 AP, 10 Hz in the presence of 80 nM folimycin, followed by a brief pulse of 50 mM NH_4_Cl to visualize the total vesicle pool.

### Visualisation of ADBE Using Electron Microscopy

Hippocampal neurons used in the electron microscopy experiment were from embryonic day 18 Sprague Dawley rat embryos. Approximately 400 cells/mm^2^ were plated on 25 mm glass coverslips coated with 1.5 µg/ml poly-D-lysine and 5 µg/ml laminin, in neurobasal medium supplemented with B-27, 0.5 mM L-glutamine and 1% penicillin/streptomycin. Glial proliferation was suppressed by addition of 1 µM cytosine-B-D arabinofuranoside at 3 days in vitro (DIV). After 22 DIV, cells were mounted in a slotted-bath imaging chamber (Warner Instruments RC-21BRFS) in 10 mg/ml horseradish peroxidase (HRP) dissolved in saline (formulation as described above). A total of 200 or 1200 1 ms pulses were delivered at either 10 Hz or 80 Hz. HRP solution was aspirated at the end of stimulation and each coverslip transferred to 2% glutaraldehyde in PBS then incubated at 37°C for 30 min. Cells were washed in 100 mM Tris (pH 7.4) then briefly incubated in 100 mM Tris/0.1% diaminobenzidine/0.2% H_2_O_2_ until reaction product formation. Cells were then stained with 1% OsO_4_ for 30 min, 2% aqueous uranyl acetate for 15 min, before dehydrating in an ethanol series and embedding in Durcupan. Sections were mounted on grids and imaged using an FEI Tecnai 12 transmission electron microscope. Nerve terminals containing HRP were analysed by counting the number of HRP-labelled endosomes per field of view. Images were analysed in Macnification (Orbicule, Inc.). HRP-labelled structures >100 nm in diameter were classified as endosomes, those <100 nm were classified as vesicles.

### Statistical Analysis

**Figure 1:**
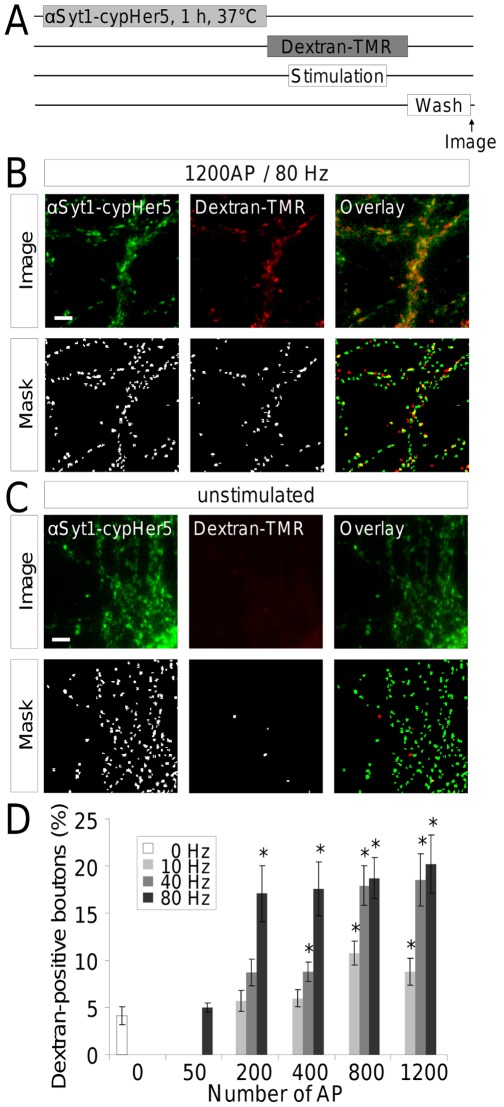
The initiation of ADBE depends on the frequency rather than the number of AP. (**A**) Experimental procedure. Functional synapses of rat hippocampal neurons were fluorescently labelled with anti-synaptotagmin1-cypHer5 (αSyt1-cypHer5) which fluoresces due to the acidic pH in the acidic vesicular lumen after endocytosis. The cells were then electrically stimulated with varying intensities (see panel (**D**)) in the presence of 50 µM dextran-TMR, which serves as a marker for bulk membrane invaginations. (**B**) Representative images of αSyt1-cypHer5 antibody-stained hippocampal neurons that were stimulated with 1200 AP, 80 Hz in the presence of dextran-TMR (“image”). Quantification of dextran-positive terminals was done by a Laplace operator based peak-detection (“mask”) followed by colocalization analysis. (**C**) Representative images of αSyt1-cypHer5 antibody-stained hippocampal neurons that were exposed to dextran-TMR without electrical stimulation. Automatically detected regions of interest are indicated in the lower panel. (**D**) Dextran-TMR positive boutons indicating terminals with bulk endosomes were quantified for varying numbers of action potentials (50, 200, 400, 800, 1200) and varying frequencies (10, 40, 80 Hz) of electrical stimulation. N = 3 to 4 experiments with 3 to 6 fields of view each; error bars represent SEM; One-way ANOVA for groups of constant numbers of AP and varying Hz: 200 AP: p<0.05; 400 AP: p>0.05; 800 AP: p<0.05; 1200 AP: p<0.01; Post-hoc test: Dunnett’s test. Asterisks indicate significant differences to unstimulated control at the 5% probability level for a 15 group comparison with a group size of 12 (critical value 2.87).

Statistical analysis was performed by MATLAB (The MathWorks Inc., Natick) or Microsoft Excel. Error bars indicate SEM unless otherwise indicated. For single group comparisons unpaired t-tests were applied (6C, D). For multiple group comparisons Tukey’s Multiple Comparison Test (Fig. 3) or a Dunnett’s test against unstimulated control (Fig. 1D) was performed. The critical value was determined for a 15 group comparison with a group size of 12 each according to [30]. In Fig. 5E the αSyt1-cypHer5 fluorescence intensity was correlated to the percentage of dextran-positive boutons. Synapses of several fields of view from 3 individual experiments (1200 AP, 40 Hz stimulations) were sorted according to their fluorescence intensities in 8 equally sized bins as described [29] and the percentage of dextran-positive terminals evaluated. Depicted are mean percentages +/− SEM. R: coefficient of determination (Spearman’s rho).

## Results

### Triggering of ADBE is Dependent on Stimulation Pattern, not Stimulation Load

ADBE is the dominant SV retrieval mode during intense stimulation in central nerve terminals [9], however little is known regarding the key physiological parameters required for its triggering. To address this, we visualized ADBE using the large fluid phase marker dextran-tetramethylrhodamine (dextran-TMR) in dispersed hippocampal neuronal cultures. Dextran-TMR specifically reports the occurrence of ADBE, since it cannot access small SVs [18], [21], [31]. During preliminary experiments we noted that dextran uptake was not confined to synaptic terminals, as glia cells also retained the reporter. To overcome this problem we incubated cultures with an anti-synaptotagmin antibody conjugated to the fluorescent probe cypHer5 (αSyt1-cypHer5) prior to dextran loading (Fig. 1A). The resultant cypHer fluorescence signal therefore enabled the identification of dextran signals specifically at synaptic sites.

When cells were stimulated with a high frequency train of action potentials (APs, 80 Hz, 15 sec) in the presence of 50 µM dextran-TMR, a clear punctate staining of synaptic terminals was observed, indicating triggering of ADBE (Fig. 1B). In contrast, under identical conditions but in the absence of stimulation, no dextran-TMR staining occurred (Fig. 1C), highlighting the activity-dependent nature of ADBE.

To determine how the interplay between stimulation intensity and stimulation load controls ADBE in hippocampal nerve terminals, AP trains of varying frequency and number were delivered in the presence of dextran-TMR. Dextran- and αSyt1-cypHer5-positive terminals were quantified using Laplace operator based peak-detection [26], with the resulting masks depicted in Figure 1. Spot detection was followed by a colocalization analysis to identify dextran-TMR-positive synaptotagmin-labelled boutons. A small number (4%) of synaptotagmin1-positive sites were also dextran-TMR-positive in the absence of stimulation. This is presumably due to either unspecific dextran-TMR binding or uptake by a constitutive fluid-phase endocytosis mode such as marcopinocytosis. Increasing both the frequency and number of action potentials (AP) resulted in an increase in the percentage of dextran-positive synaptic terminals (Fig. 1D). No differences in the intensities of dextran-TMR puncta were apparent, in agreement with previous studies [31]. When stimulation load (AP number) was kept constant, dextran-TMR uptake scaled with increasing stimulation frequency across all stimulation loads (Fig. 1D). Conversely when stimulation frequency (Hz) remained constant, dextran-TMR uptake did not display such a clear relationship. For example, dextran-TMR uptake occurred in approximately 20% of synaptotagmin-positive sites during stimulation trains of 80 Hz regardless of duration, with the exception of 50 APs (Fig. 1D). For some stimulation paradigms however, AP numbers seem to play a role: For the 40 Hz stimulations, a marked increase in ADBE occurred at 800 and 1200 AP (17,9% and 18.5%). Similarly low frequency stimulation trains (10 Hz) significantly increased ADBE during 800 and 1200 AP (10.8% and 8.8% respectively) compared to unstimulated control (Fig. 1D). Thus the major determinant in triggering of ADBE is the stimulation intensity (Hz) and not the stimulation load (AP number).

### Validation of Dextran-TMR as a Tool to Study ADBE

**Figure 2:**
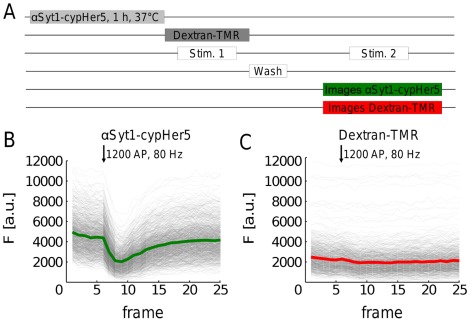
Dextran-TMR cannot be released from synaptic terminals by exocytosis. (**A**) Experimental procedure. Functional synapses of rat hippocampal neurons were fluorescently labelled with αSyt1-cypHer5. Dextran-TMR was subsequently loaded with 1200 AP, 80 Hz (Stim. 1). After removing extracellular dextran-TMR (wash), images in the Cy5- and TRITC channels were acquired during a second electrical stimulation with 1200 AP, 80 Hz (Stim 2). (**B**) αSyt1-cypHer5 fluorescence intensity over time for a representative experiment (>900 synapses). A.u., arbitrary units. (**C**) The corresponding dextran-TMR fluorescence intensity over time during the second stimulation was unaltered by the electrical stimulation. A.u., arbitrary units.

We next determined whether accumulated dextran-TMR could be subsequently released from synaptic terminals by high frequency stimulation. No release of the reporter was observed under these conditions (Fig. 2). Thus dextran-TMR specifically reports the generation of bulk endosomes, without dilution of the fluorescent signal from either 1) direct fusion of endosomes with the plasma membrane or 2) generation of dextran-TMR-loaded SVs from these endosomes in agreement with previous studies [18], [31]. Further evidence that large dextrans accurately report ADBE comes from studies, where the number of dextran puncta visualized using fluorescence microscopy closely correlate with the number of HRP-labelled endosomes (but not HRP-SVs) in nerve terminals across a range of different stimulation trains [32]. Manipulations that specifically inhibit ADBE cause a parallel reduction in both dextran uptake and HRP endosome number, but show no effect on HRP-labelled SVs [33], [34]. Moreover, large dextrans have been found to be associated with endosomal markers by fluorescence microscopy of fibroblasts [35] and finally ultrastructural analysis of photoconverted FITC-dextran confirmed the localization of dextrans in endosomal structures [36].

### Electron Microscopy Confirms the Frequency-dependence of ADBE

**Figure 3:**
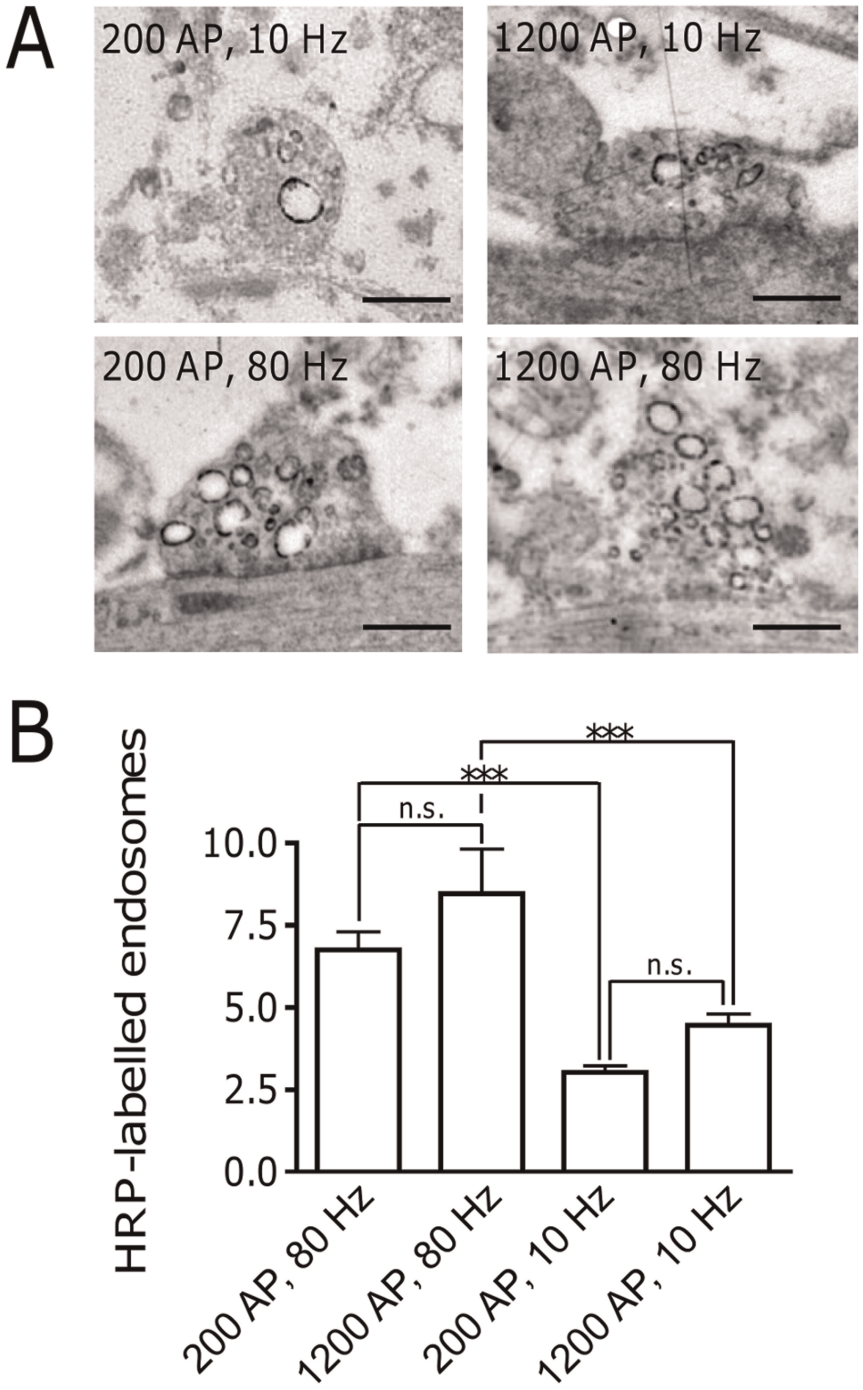
Electron microscopy confirms that the initiation of ADBE depends mainly on the frequency of electrical stimulation. (**A**) Representative electron microscopy images of HRP-labelled endosomes by electron microscopy. Hippocampal neurons were electrically stimulated with 200 AP at 10 Hz, 200 AP at 80 Hz, 1200 AP at 10 Hz and 1200 AP at 80 Hz, respectively, in the presence of 10 mg/ml HRP. Scale bar, 500 nm. (**B**) Quantification of HRP-labelled endosomes by electron microscopy. Data presented is the average of 3 independent experiments ± SEM. Indicated statistical significances are from a one way ANOVA α = 0.05 followed by Tukey’s Multiple Comparison Test: n.s., not significant; ***p<0.001.

To confirm that stimulation frequency is the major factor in ADBE triggering, we measured ADBE in single nerve terminals by monitoring the uptake of the fluid phase marker horse radish peroxidase (HRP). Synaptic terminals undergoing ADBE can be visualized by the appearance of electron-dense endosomes, after conversion of HRP [9], [33], [34], [37]. A maximal stimulus of 1200 APs delivered at 80 Hz resulted in a robust incorporation of HRP into synaptic endosomes (Fig. 3) confirming ADBE had occurred. A similar number of HRP-labelled endosomes was also observed when frequency was maintained at 80 Hz but stimulation load was reduced to 200 AP (Fig. 3). The number of HRP-labelled endosomes decreased dramatically when stimulation frequency was reduced to 10 Hz, even though stimulation load remained constant (200 or 1200 APs). These results confirm our previous observations with dextran-TMR, highlighting that stimulation intensity (Hz) is the major determinant of ADBE triggering in hippocampal synaptic terminals.

A potential explanation for the increased prevalence of ADBE after stimulation with 200 AP, 80 Hz when compared to 1200 AP 10 Hz is that an increased amount of exocytosis may have occurred during the 80 Hz stimulation protocol. To test this we determined the extent of exocytosis evoked by either 200 AP (80 Hz) or 1200 AP (10 Hz) using the genetic reporter superecliptic synaptopHluorin. We found that 200 AP, 80 Hz released 14.0±2.0% of the SV pool, while 1200 AP, 10 Hz released 29.5±3.7% (Fig. S1). Thus the increased occurrence of ADBE at high stimulation frequencies is not directly dependent on the prior extent of exocytosis.

### The Prevalence of ADBE Increases with SV Recycling Pool Size

**Figure 4:**
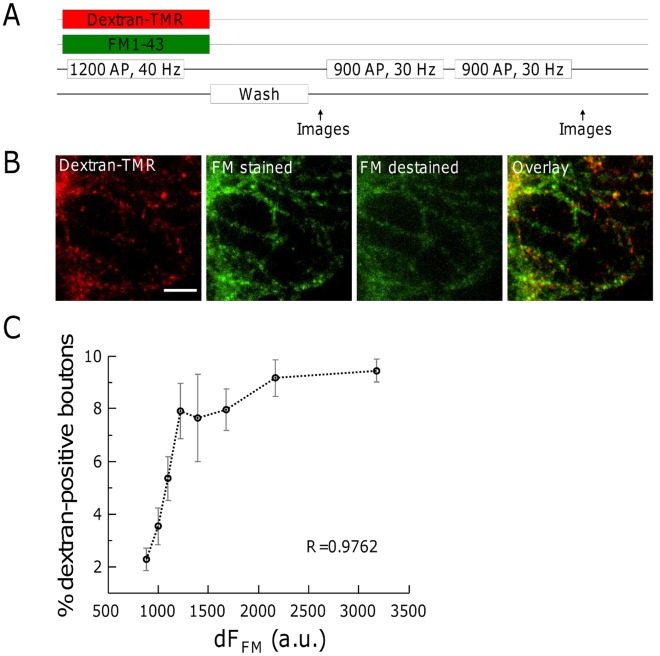
Formation of bulk endosomes correlates with the recycling pool size. (**A**) Experimental procedure. Hippocampal neurons were simultaneously loaded with 50 µM dextran-TMR and 2.5 µM FM1-43 by a 1200 AP, 40 Hz electrical stimulation. After washing away extracellular dye, images for FM1-43 and dextran-TMR were acquired. Complete destaining of FM1-43 was done by two subsequent electrical stimulations (2×900 AP, 30 Hz). (**B**) Representative images of dextran-TMR and FM1-43 labelled hippocampal neurons. Scale bar, 10 µm. (**C**) The number of dextran-TMR-positive spots increases with dF(FM). All synapses in 5 fields of view of each of 3 individual experiments were evaluated by sorting according to their fluorescence intensity and subsequent binning into 8 groups containing equal numbers of terminals. The percentage of dextran-positive terminals in each group was determined. Displayed is the mean of all fields of view. R, spearman’s rho.

**Figure 5:**
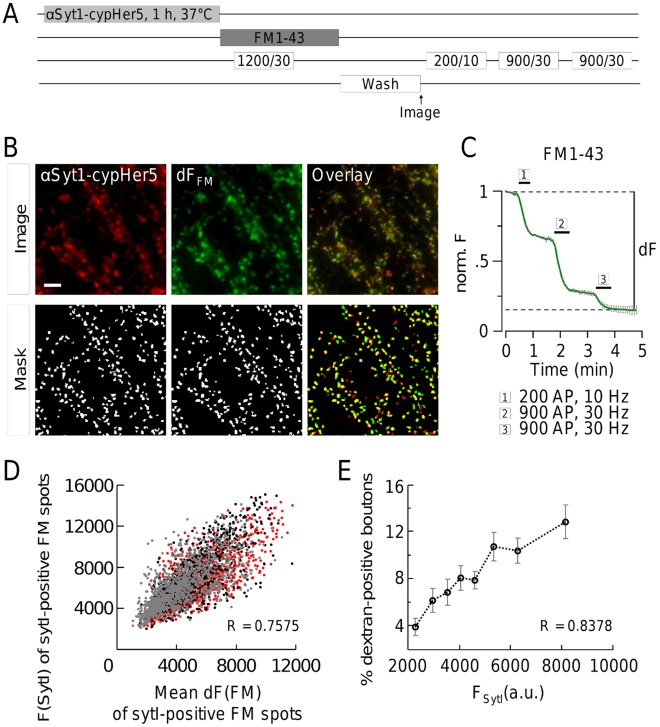
Formation of bulk endosomes increases with vesicle number per synapse. (**A**) Experimental procedure. Functional synapses of rat hippocampal neurons were fluorescently labelled with αSyt1-cypHer5. The cells were then electrically stimulated with 1200 AP, 30 Hz in the presence of 1 µM FM1-43, to fully load synaptic vesicles with FM dye. Complete destaining was done by three subsequent electrical stimulations (200 AP, 10 Hz, 2×900 AP, 30 Hz). (**B**) Representative images of αSyt1-cypHer5 antibody-stained hippocampal neurons that were loaded with FM1-43 (“image”). Quantification was done by a Laplace operator based peak-detection (“mask”). (**C**) Normalized FM1-43 fluorescence intensity over time. Electrical stimulation was done as described in (A) and fluorescence differences (dF) calculated for every synapse. N = 3 experiments, n>1000 synapses each, Error bars represent SEM of 3 individual experiments. (**D**) Correlation of αSyt1-cypHer5 fluorescence and the fluorescence difference of FM1-43. Each dot represents a single bouton, black, grey and red colors indicate individual experiments (N = 3); n>1000 synapses each; R, spearman’s rho. (**E**) The number of dextran-TMR-positive spots increases with αSyt1-cypHer5 fluorescence. All synapses in 4 fields of view of each of 3 individual experiments were evaluated by sorting according to their fluorescence intensity and subsequent binning into 8 groups containing equal numbers of terminals. The percentage of dextran-positive terminals in each group was determined. Displayed is the mean of all fields of view. R, spearman’s rho.

ADBE only occurs in a subset of synaptic terminals (Fig. 1). Therefore we next questioned if any physiological characteristics potentially predict which synaptic terminals undergo ADBE. One potential predictor could be the size of the total SV recycling pool. This has been quantified previously by loading synaptic terminals with a styryl dye such as FM1-43 during a saturating stimulus train. This loading is then followed by a similar saturating stimulus to evoke complete dye unloading [24], [28]. To investigate the relation of synapse size and initiation of ADBE, we loaded FM1-43 and dextran-TMR in parallel with 1200 AP, 40 Hz. We observed a positive correlation (R = 0.9762) between SV recycling pool size and initiation of ADBE (Fig. 4). However, FM1-43 also labels ADBE [23] meaning that the quantification of SV pool size by dye unloading could be skewed by retention of dye in bulk endosomes. Therefore, we determined whether the αSyt1-cypHer5 signal was a more suitable method to determine the size of the total SV recycling pool. Cells were incubated for 1 h with αSyt1-cypHer5 and then FM1-43 was loaded with 1200 AP at 30 Hz (Fig. 5A). After washing away extracellular FM1-43, a punctate pattern was observed for both FM1-43 and αSyt1-cypHer5 staining (Fig. 5B). Three subsequent stimulations evoked a complete unloading of the SV recycling pool, enabling the determination of the fluorescence difference dF(FM) for every synaptic terminal (Fig. 5C). We found that αSyt1-cypHer5 fluorescence intensity correlates well (R = 0.7575) with the dF(FM) values from FM1-43-stained boutons (Fig. 5D). Thus, αSyt1-cypHer5 fluorescence intensity is an appropriate measure of total SV pool size.

Having established αSyt1-cypHer5 fluorescence intensity as a reporter of total SV recycling pool size, we correlated this value with the number of dextran-TMR-positive terminals. We observed a strong correlation between the percentage of dextran-TMR-positive boutons and synaptic terminals with a large total SV recycling pool (R = 0.8378) (Fig. 5E). Thus the larger the size of the recycling pool in synaptic terminals, the greater the probability that ADBE will be triggered during high intensity stimulation.

### Inhibitory Synaptic Terminals have a Greater Incidence of ADBE

**Figure 6:**
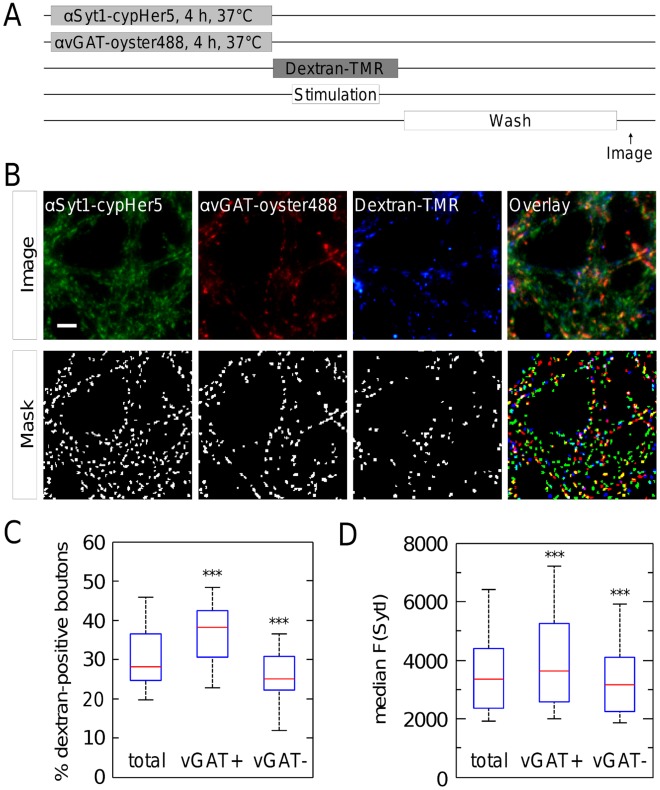
Inhibitory synapses preferentially form bulk endosomes and are slightly larger as compared to excitatory synapses. (**A**) Experimental procedure. Functional synapses of rat hippocampal neurons were fluorescently labelled with αSyt1-cypHer5. In parallel, inhibitory synapses were identified with an anti-vGAT (vesicular GABA transporter) antibody, labelled with the green fluorescent dye Oyster488®. The cells were then electrically stimulated with 1200 AP, 40 Hz in the presence of 50 µM dextran-TMR. (**B**) Representative images of αSyt1-cypHer5 and anti-vGAT-oyster488® antibody-stained hippocampal neurons that were exposed to dextran-TMR during the electrical stimulation (“image”). Quantification was done by a Laplace operator based peak-detection (“mask”). (**C**) vGAT-positive synapses showed a larger percentage of dextran-TMR-staining than vGAT-negative ones or the total of synapses. The boxplot indicates median (red line), 25th and 75th percentiles (blue box) and extreme values (whiskers). Wilcoxon signed rank test, ***p<0.001. N = 4 experiments with 4 to 7 fields of view each; (**D**) vGAT-positive synapses were larger than vGAT-negative ones or the total of synapses.

Specific subsets of inhibitory interneurons are characterized by high rates of activity [38], suggesting that they may utilize ADBE to compensate for this elevated activity. Therefore, we next determined whether ADBE was more prevalent in inhibitory synaptic terminals in our hippocampal cultures. To identify inhibitory synapses, we marked GABAergic neurons with a fluorescently-labelled antibody against the luminal domain of the vesicular GABA transporter (αvGAT-oyster488) [39] simultaneously with the αSyt1-cypHer5 incubation. Then we induced ADBE with 1200 AP at 40 Hz in the presence of dextran-TMR as before (Fig. 6A). We identified a subpopulation of the αSyt1-cypHer5-positive boutons to be vGAT-positive within our cultures (34.3±10.7%), which is in line with findings from other studies [40]. ADBE could be visualized by dextran-TMR-uptake in both vGAT-positive and –negative synaptic terminals (Fig. 6B). Interestingly, the vGAT-positive synaptic terminals displayed a significantly higher percentage of dextran-TMR-labeling compared to the total number of synapses as defined by the αSyt1-cypHer5 signal (p<0.001) (Fig. 6C). Conversely, vGAT-negative synaptic terminals showed significantly less dextran-TMR-uptake (p<0.001). Thus GABAergic synaptic terminals are more likely to utilize ADBE as an endocytosis mechanism than non-GABAeric terminals.

Since SV recycling pool size is a predictor of whether synaptic terminals utilize ADBE (Fig. 5E), we determined whether this parameter differed between vGAT-positive and vGAT-negative synapses. vGAT-positive synapses displayed a significantly larger median αSyt1-cypHer5 fluorescence intensity when compared against all synaptic terminals (p<0.001) (Fig. 6D). Reciprocally, vGAT-negative synapses displayed a significantly lower median intensity (p<0.001). Since αSyt1-cypHer5 fluorescence intensity is an accurate reporter of SV recycling pool size (Fig. 5D), we conclude GABAergic synaptic terminals have on average a larger SV recycling pool, potentially explaining their higher propensity to initiate ADBE (Fig. 4C, 5E).

## Discussion

In this manuscript we have shown that 1) ADBE is triggered by high intensity stimulation in small central nerve terminals, 2) this triggering is dependent on stimulation frequency rather than stimulation load, 3) synaptic terminals with larger SV recycling pools have a greater probability of utilizing ADBE, and 4) GABAergic nerve terminals have on average a larger recycling SV pool and have a higher probability of utilizing ADBE. Thus ADBE is triggered by physiological stimulation in a key population of synaptic terminals, implicating it as critical modulator of hippocampal physiology.

### Heterogeneous Triggering of ADBE

To investigate the conditions for the initiation of ADBE we chose an optical approach by using fluorescently labelled dextran. In order to increase the specificity of this marker, we costained hippocampal synapses with an anti-synaptotagmin1 antibody. αSyt1-cypHer5 is a specific marker for functional synapses and enables the estimation of synapse size, because the number of synaptotagmin1 molecules per vesicle shows a low intravesicular variation with 7 or 8 molecules per vesicle [41].

As revealed in ultrastructural studies the site of endocytosis is not identical with the active zone [19]. Therefore, we considered a dextran signal to be synaptic when it colocalized with the αSyt1-cypHer5 fluorescence in a 1 µm radius. Inevitably, this will result in false-positive counts of ADBE and explains the basal ADBE rate of ∼4% even in the absence of electrical stimulation. Considering this 4% background, stimulations with either low frequency (200 AP, 10 Hz; 400 AP, 10 Hz) or high frequency for short durations (50 AP, 80 Hz) did not induce bulk endosomes. However, increasing the duration of stimulation with 10 Hz (800 AP, 10 Hz; 1200 AP, 10 Hz), seemed to provoke some ADBE (10.8% and 8.8%).

We show that the frequency of stimulation is a much stronger predictor for the initiation of ADBE than simply the number of APs. This finding is in agreement with previous studies in primary cultures of cerebellar neurons [9]. The coupling of ADBE to stimulation frequency rather than stimulation load suggests that the key challenge for the synaptic terminal is not in handling the overall extent of SV exocytosis, but rather the extent of SV exocytosis within a defined period of time. This is highlighted by our finding that ADBE is more prevalent in synapses that are challenged with short trains of high frequency APs, even though this protocol evoked only 50% of the extent of exocytosis compared to longer trains of low frequency stimulation (Fig. S1). This challenge arises because CME has a limited capacity to retrieve SVs in the short term [25]. It also suggests that the mechanism for triggering of ADBE is independent of the molecular apparatus that mediates SV exocytosis. How is ADBE coupled to stimulation intensity? The proposed triggering mechanism for ADBE is dephosphorylation of the GTPase dynamin I by the calcium-dependent protein phosphatase calcineurin. Importantly this event is dependent on the stimulation frequency but not the stimulation load [33]. Thus the pattern of neuronal activity is the critical determinant in the triggering of ADBE, and not the overall extent of SV exocytosis.

We report that synaptic terminals with large SV recycling pool sizes appear more likely to perform ADBE. This heterogeneity might be explained by the non-linear relation between volume (corresponding to the SV content of a synaptic terminal) and the synaptic membrane surface [29]. Thus synapses that contain a large SV recycling pool will incorporate proportionately more SV membrane into the nerve terminal membrane and thus will need to retrieve membrane more efficiently.

### Physiological Relevance of ADBE

The occurrence of ADBE in hippocampal neurons has been questioned [12], [21], [42]. However we found that approximately 20% of all functional synaptic terminals underwent ADBE, with the figure even higher for inhibitory neurons. A previous study determined the prevalence of ADBE in cerebellar granule neuron terminals found that ADBE occurred in approximately 35% of all functional nerve terminals [31], suggesting that the occurrence of ADBE may vary depending on the specialization of the neuron. Cerebellar granule neurons receive high frequency (100–200 Hz) bursts of input from mossy fibres [43], suggesting the higher utilization of ADBE is a strategy to allow these neurons to adapt to these intense periods of stimulation. The higher observed prevalence of ADBE in GABAergic neurons may also be explained by the fact that many inhibitory interneurones have high rates of activity [38]. Thus the prevalence of ADBE in different neuronal subtypes may reflect different adaptations to their physiological function.

The fact that a significant subpopulation of excitatory hippocampal neurons utilizes ADBE to retrieve SV membrane suggests that ADBE will form a major contribution towards hippocampal neurotransmission and function. In agreement, inhibition of presynaptic glycogen synthase kinase 3 (which is essential for ADBE) resulted in a relief of synaptic depression in hippocampal slices [34]. Thus manipulation of the extent of ADBE may impact on LTP and memory generation and in pathological terms may either exacerbate or diminish seizure activity.

## Supporting Information

Figure S1 **The higher incidence of ADBE during high frequency stimulation is not due to a higher number of exocytosed synaptic vesicles.** (**A**) Experimental procedure. SynaptopHluorin-expressing hippocampal neurons were stimulated with either 200 AP, 80 Hz, or 1200 AP, 10 Hz in the presence of 80 nM folimycin. To determine the total pool of SV, a short pulse of 50 mM NH_4_Cl was applied. (**B**) Representative images of synaptopHluorin-expressing hippocampal neurons before and during electrical stimulation and the NH_4_Cl pulse. Scale bars, 10 µm. (**C**) SynaptopHluorin fluorescence intensity over time was normalized to the fluorescence intensity during NH_4_Cl application. N(200 AP, 80 Hz) = 5 experiments, N(1200 AP, 10 Hz) = 5 Experiments. n>400 synapses each, (**D**) Quantification of the percentage of exocyosed SV from the total SV pool after stimulation with 200 AP, 80 Hz and 1200 AP, 10 Hz. Error bars represent SEM of 5 individual experiments.
(TIF)
