# Supplementary figures and images for: Key Physiological Parameters Dictate Triggering of Activity-Dependent Bulk Endocytosis in Hippocampal Synapses
Source: PLoS One. 2012 Jun 4;7(6):e38188. doi: 10.1371/journal.pone.0038188 (PMC3366995; doi:10.1371/journal.pone.0038188)

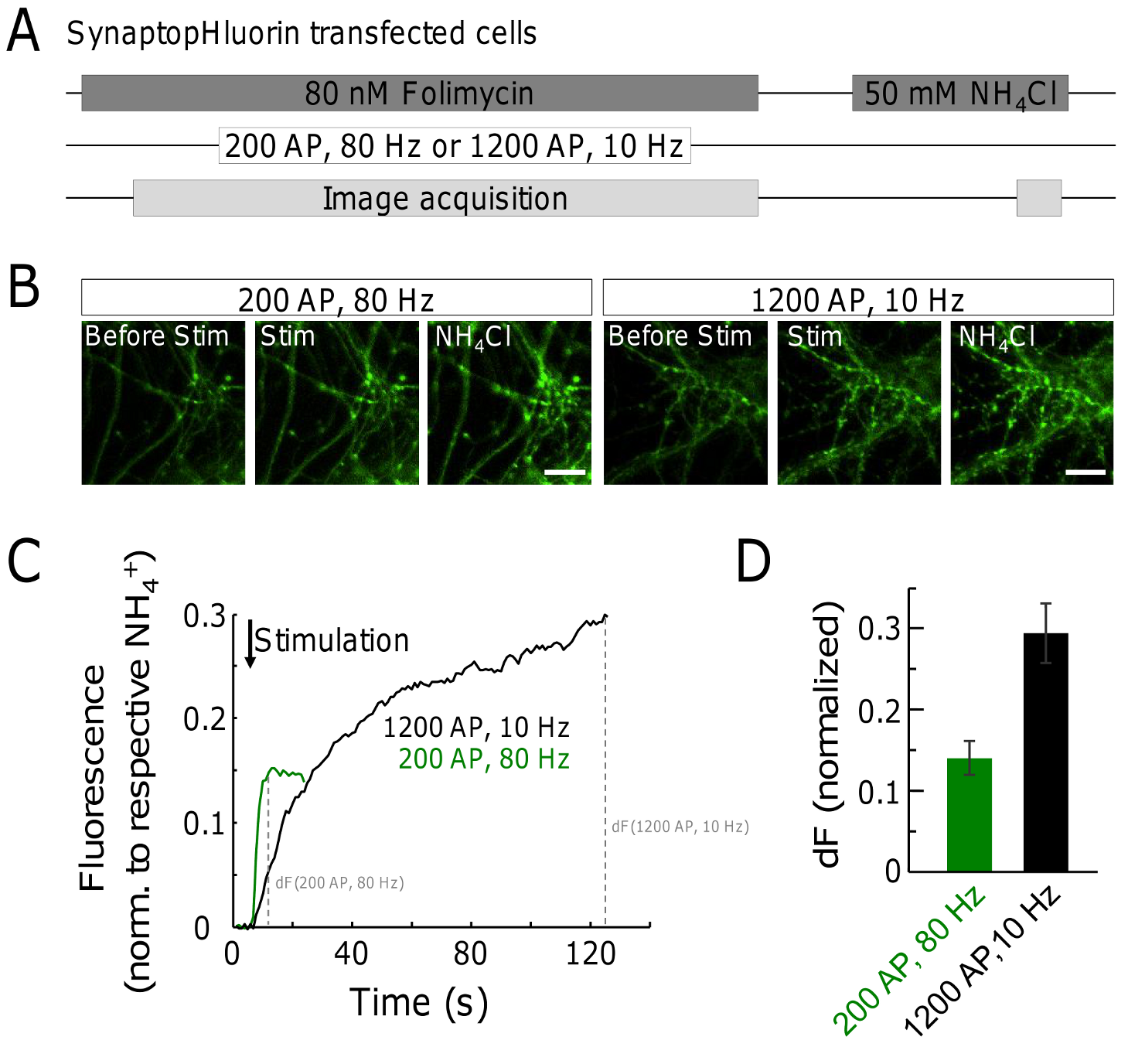

Supplement: Figure S1 — The higher incidence of ADBE during high frequency stimulation is not due to a higher number of exocytosed synaptic vesicles. (A) Experimental procedure. SynaptopHluorin-expressing hippocampal neurons were stimulated with either 200 AP, 80 Hz, or 1200 AP, 10 Hz in the presence of 80 nM folimycin. To determine the total pool of SV, a short pulse of 50 mM NH4Cl was applied. (B) Representative images of synaptopHluorin-expressing hippocampal neurons before and during electrical stimulation and the NH4Cl pulse. Scale bars, 10 µm. (C) SynaptopHluorin fluorescence intensity over time was normalized to the fluorescence intensity during NH4Cl application. N(200 AP, 80 Hz) = 5 experiments, N(1200 AP, 10 Hz) = 5 Experiments. n>400 synapses each, (D) Quantification of the percentage of exocyosed SV from the total SV pool after stimulation with 200 AP, 80 Hz and 1200 AP, 10 Hz. Error bars represent SEM of 5 individual experiments. (TIF) [file pone.0038188.s001.tif]
